# 

**Published:** 1918-01

**Authors:** 


### TABLE OF CONTENTS

Double, Recurrent and Bilateral Tubal Pregnancies ----- 285

Conservative Operation of the Ovaries --------------- 294

Heredity ------------------------------------------ 300

Women in Railway Service as Viewed from a Surgical Standpoint; Pertinent to the World War ---------- 309

Medical Items -------------------------------------- 314

